# Work till old age: an analysis of self-employment’s impact on depression among the older adults in China

**DOI:** 10.3389/fpsyt.2024.1426336

**Published:** 2024-10-17

**Authors:** Li He, Jinxu Zhao, Man Li, Zhiyong Song, Yanling Ma, Zhixiong Yang

**Affiliations:** ^1^ Zhongnan University of Economics and Law, School of Philosophy, Wuhan, Hubei, China; ^2^ Ocean University of China, School of Marxism, Qingdao, Shandong, China

**Keywords:** self-employed older adults, retiree, depression level, urban-rural differences, China

## Abstract

**Background:**

Depression is a significant risk factor affecting the mental health of older adults. In the context of accelerated population aging and the policy of “delayed retirement,” self-employment has become an important alternative for older adults. Thus, studying the difference in depression levels between self-employed older adults and retirees, along with the mechanisms behind these differences, has emerged as a crucial theoretical and practical issue.

**Methods:**

This research, based on panel data from the China Health and Retirement Longitudinal Study for the years 2011, 2013, 2015, and 2018, employed fixed-effect, instrumental variable, mediation models to analyze the difference in depression levels between self-employed older adults and retirees, as well as the mediating mechanisms involved.

**Results:**

The findings indicate that self-employed older adults have lower levels of depression than retirees. The results of the mediating mechanism analysis suggest that self-employment can indirectly lower the depression levels of older adults by frequent social participation and greater life satisfaction. However, heterogeneity analysis revealed significant urban–rural differences and different types of self-employment in the impact of self-employment on the depression levels of older adults.

**Conclusions:**

The results of this study are of great significance for enhancing the mental health of older adults and provide empirical support for China and other developing countries in formulating more effective aging policies and building a more beneficial aging society.

## 1 Introduction

The World Health Organization defines depression as a common mental disorder.^1^ Studies have shown that depression significantly impacts the quality of life of older adults and may become a critical risk factor for inducing suicide (1, 2). Globally, about 5.7% of adults over 60 suffer from depression.^2^ Research has also revealed that approximately one-third of retirees are troubled by depression (3), making depression an increasingly significant risk factor for disability and death among older adults (4).

However, it is noteworthy that with the acceleration of the aging process, more and more older adults choose to engage in self-employment. This may be because, compared to employed labor, the flexibility of self-employment (5), financial considerations after retirement (6), and the opportunity for self-employment to keep older adults active make self-employment more attractive to them (7). As of 2020, about 18% of workers aged 50–64 in the European Union were self-employed, and the proportion of self-employed people aged 65–69 increased to 39%.^3^ As of 2019, 46% of Americans aged 65–69 were self-employed, indicating that an increasing number of older adults are choosing self-employment.^4^ Moreover, the depression levels among this group of self-employed individuals are generally lower than those who are not self-employed (8, 9). Additionally, compared to employed older adults, self-employed older adults experience greater variability in their work environments and tasks, making them more representative in discussions about the diversity of mental health. Therefore, it is crucial to deeply understand the depression conditions of self-employed older adults and the underlying mechanisms affecting them.

Self-employment is one of the significant options for older adults to continue working after retirement (10). Thus, when exploring the depression levels of self-employed older adults, it is also essential not to overlook the depression conditions of those who continue to work after retirement. However, there is controversy in academia regarding the relationship between continuing to work past retirement age and depression levels among older adults. Some researchers have found that continuing to work past retirement age may not be beneficial for improving the mental health of older adults, especially in situations where they are forced to work out of economic necessity or in fields other than their preretirement-age occupations (11, 12), potentially increasing their risk of depression. On the other hand, other studies have shown that continuing to work past retirement age can have positive effects on older adults (13, 14), such as enhancing their social activities and helping them obtain more social support (15). Given that many older adults continue working past retirement age through self-employment (10), the depression levels of self-employed older adults must be explored.

Furthermore, previous studies have shown an uneven regional distribution, with most studies focusing on developed countries and regions rather than developing countries. As the largest developing country in the world, China has a considerably older population. By 2020, the number of people aged 60 and above in China had reached 264 million, accounting for 18.7% of the total population. Notably, with societal changes, an increasing number of older Chinese adults are choosing to engage in self-employment (9). However, there is relatively little research on depression among older adults in China, especially among self-employed older adults. Based on this, this study targeted self-employed older adults and retirees as research subjects and delved into two questions: Is there a difference in depression levels between self-employed older adults and retirees? If so, what factors contribute to this difference?

To find the answers to the aforementioned questions, this study utilized sample data from China, with the labor status of older adults as the independent variable and depression level as the dependent variable. It employed fixed-effect and mediation effect models to explore the difference in depression levels between self-employed older adults and retirees and further analyzed the mediating mechanisms of social interaction and life satisfaction, among others. To ensure the most accurate analysis results, this study also referenced the instrumental variable (IV) model to address potential issues of reverse causality and endogeneity in the experimental data. The data used in this study came from the China Health and Retirement Longitudinal Study (CHARLS) conducted in 2011, 2013, 2015, and 2018.

## 2 Literature review and research hypotheses

### 2.1 Literature review

The self-employment activities of older adults fall within the interdisciplinary fields of psychology, socioeconomics, and gerontology, encompassing not only career choices past retirement age but also the psychological health and social interactions of older adults. Previous studies have primarily focused on the impact of self-employment on the social and economic lives of older adults, with relatively fewer studies exploring the relationship between self-employment and older adults’ psychological health, especially in terms of how self-employment affects the depression levels of older adults.

#### 2.1.1 Review of the Concept of Self-Employment

The self-employed are people who operate their own businesses or services independently. They are economically independent, typically not employed by others but instead manage and operate their own businesses or services. This group mainly includes individual operators, small business owners, freelancers, and others. The OECD categorizes the self-employed into three types: sole proprietors without employees, employers with employees, and unpaid family workers (16). Self-employed older adults are typically those who have reached or exceeded the traditional retirement age but choose to continue or start self-employment activities (17). These include people who may have been engaged in self-employment for a long time and those who transition from traditional employment to self-employment past retirement age. This study primarily focused on older adults defined as self-employed at the time of the survey. Due to data limitations, we were unable to further distinguish between older adults with continuous self-employment and those who transitioned from other forms of employment to self-employment.

#### 2.1.2 Factors influencing depression in older adults

With advancing age, people undergo lifestyle changes, often accompanied by negative emotions such as anxiety and depression (18). The causes of these emotions vary, covering personal, familial, and social levels. On a personal health level, physical deterioration, increased susceptibility to diseases, and cognitive decline lead to pessimistic emotions in older adults, and even life-threatening behaviors such as suicide (19). A considerable body of research suggests that a lack of familial care can further exacerbate the depressive behaviors of older adults. In particular, insufficient care and companionship from their children can weaken their connection and intimacy with their family members, thereby increasing their risk of depression (20). Additionally, social factors play a crucial role in the psychological health of older adults. Previous studies have found that a lack of social support is a significant cause of high depression rates among them (21). Notably, however, few studies have focused on the behavioral factors linked with working past retirement age, especially the psychological condition of older adults in self-employment scenarios.

#### 2.1.3 Self-employment and depression among older adults

Self-employment is increasingly becoming the primary work choice for retired older adults (9). It is necessary to examine the specific psychological manifestations of older adults working in a self-employed context. However, previous studies have not reached a consensus on the relationship between self-employment and depression among older adults. Some scholars believe that self-employment may increase economic pressures and life balance challenges for older adults, thereby negatively affecting their psychological health (22, 23). Other researchers have focused on retirement as a critical life stage, suggesting that the impact of self-employment on the depression levels of older adults varies significantly according to retirement age, with those who choose to engage in self-employment beyond the standard retirement age potentially experiencing higher levels of depression. Conversely, some researchers lean toward the positive effects of self-employment on the physical and mental health of older adults. They have pointed out that compared to retired and out-of-work people, people who remain active in the labor market, especially those engaged in self-employment, have higher work autonomy and a higher sense of fulfillment (23, 24). They also exhibit lower levels of depression tendencies (25).

#### 2.1.4 Exploring the Mechanism of Self-Employment Impacting Depression in Older Adults

Previous studies have found that the relationship between older-adult self-employment and depression is influenced by the dual mediation effects of economic and noneconomic factors (26). Economically, Samuels and Stavropoulou (27) noted that the income increase resulting from self-employment after retirement plays a mediating role in improving the depressive conditions of older adults (28, 29). Regarding noneconomic factors, enhanced job satisfaction, autonomy, and a sense of purpose brought about by self-employment have positive impacts on the depression levels of older adults (23, 30). Furthermore, previous studies that examined the roles of age and gender as moderating variables found that the positive impact of self-employment on depression diminishes with age (31), and that female self-employed older adults may experience lower levels of depression than their male counterparts (32). Despite these findings, the existing research still shows a lack of exploration into the mediating mechanisms of self-employment and depression levels in older adults, especially lacking an in-depth analysis of social interaction and life satisfaction. In terms of heterogeneity analysis, the differences in residential locations and *hukou* ^5^ of older adults have been overlooked.

#### 2.1.5 Activity Theory and Its Impact

Activity theory opens a theoretical window for addressing the aforementioned research gaps. It is a psychosocial theory about aging proposed by Robert Havighurst in 1961 in response to disengagement theory, advocating that social participation and activity during old age are key to achieving positive aging and emphasizing the importance of maintaining productive activities and attitudes from middle age (33). Early research on this theory explored the positive roles of social engagement, physical activities, and community participation on the life satisfaction and psychological health of older adults. However, despite significant progress in the research on older adults’ psychological health using activity theory, some theoretical gaps remain, especially in applying the theory to occupational activities, such as the study of the relationship between self-employment and depression among older adults.

In summary, existing research has limitations, and this study aimed to address these shortcomings and make innovations. First, this study focused on the relationship between self-employment and depression in older adults, delving into how the former affects the latter. Second, this study employed panel data for empirical analysis, overcoming the limitations of using cross-sectional data in previous research, and establishing causal relationships while identifying correlations. Third, this study thoroughly considered the heterogeneity of older adults, paying attention to the differences in urban–rural residency and *hukou*. Fourth, guided by activity theory, this study explored the impact of self-employed occupational activities on the psychological health of older adults. Additionally, it posited that under resource-limited conditions, self-employment could serve as an effective form of social participation, promoting the psychological health of older adults and thus supplementing activity theory.

### 2.2 Research hypotheses

The concept of “role substitution,” one of the key elements of activity theory, plays a significant role in explaining the psychological health and sense of well-being of older adults. It is crucial for their mental health (33). Self-employment, as a form of active engagement in work, can provide older adults with opportunities for new social roles. Compared to retirees, self-employed older adults not only engage in daily activities and carry out daily responsibilities but also maintain their social status and identity, thereby preserving a healthy psychological state (34), which aligns with the “role substitution” concept of activity theory. Therefore, this study hypothesized that self-employed older adults would exhibit lower levels of depression than retirees due to the continuation of their social roles past retirement age. Consequently, the following hypothesis was proposed:

Hypothesis 1: Self-employed older adults have lower levels of depression than retirees.

According to activity theory, active social participation and interpersonal interactions are key factors in the well-being and psychological health of older adults (35). Self-employment, as an important avenue for the reemployment of older adults, can provide more opportunities for social interaction. Self-employed older adults often need to maintain contact with clients, partners, and other self-employed people. Such social interactions contribute to the establishment and maintenance of their social support networks, enhance their sense of being valued (30), and have a positive effect on their psychological state. Furthermore, activity theory suggests that engagement in activities is positively correlated with life satisfaction (33). By continuing to work, self-employed older adults enhance their autonomy and identity, regain a sense of self-worth, and fulfill their spiritual needs for self-actualization (36). This continued engagement and sense of identity promote a positive perception of life, leading to increased life satisfaction. High life satisfaction is associated with lower levels of depression (37). Hence, the following hypotheses were proposed:

Hypothesis 2a: Social interaction is a mediating mechanism for lower levels of depression among self-employed older adults.

Hypothesis 2b: Life satisfaction is a mediating mechanism for lower levels of depression among self-employed older adults.

Activity theory emphasizes the importance of the environment and social participation in the psychological health of older adults (33). Older adults who engage in social activities are likely to exhibit better psychological health and lower symptoms of depression. In rural areas, where public infrastructure improvements are slow and access to public services is limited (38), older adults participate less in social activities and experience a stronger sense of relative poverty and abandonment (39). In this context, self-employment as a form of occupation provides a way for rural older adults to meet their needs for social participation, thereby significantly lowering their depression levels. Conversely, in urban settings, retirees typically enjoy more diverse opportunities for social engagement (20), and these existing supports make the unique benefits of self-employment less pronounced. Furthermore, in China, the *hukou* system has a profound impact on an individual’s social welfare, such as pensions and healthcare services. Compared to those with urban *hukou*, older adults with rural *hukou* have lower pensions and limited access to medical services (40). The disparities in welfare make rural *hukou* holders face more economic pressures and health risks. Engaging in self-employment can increase their income sources, better meet their own needs, improve their quality of life, and gain more social support and personal achievement (41), thereby achieving a more significant reduction in depression levels.

Hypothesis 3a: The reduction in depression levels is more significant in self-employed older adults living in rural areas compared to those in urban areas.

Hypothesis 3b: The reduction in depression levels is more significant in self-employed older adults with rural *hukou* compared to those with urban *hukou*.

Self-employment, as an important form of social activity, provides older adults with opportunities for financial independence and active social roles, effectively alleviating feelings of psychological isolation and depression. However, the impact of different types of self-employment on the mental health of older adults may vary (42). Agricultural self-employment, by offering meaningful physical activity, frequent community interactions, and social support (43), helps to reduce anxiety and depression. In contrast, non-agricultural self-employed individuals may lack similar mental health-promoting factors in their work environment and activities. Non-agricultural self-employed individuals often engage in repetitive and high-pressure work in urban or town settings, which may be accompanied by fewer opportunities for social interaction and a greater sense of loneliness (44). Therefore, the positive effects of non-agricultural self-employment on the mental health of older adults may be limited or diminished. Accordingly, the following hypothesis was proposed:

Hypothesis 4: Compared to non-agricultural self-employed older adults, agricultural self-employed older adults exhibit significantly lower levels of depression than retirees.

## 3 Data and methods

### 3.1 Data

The data employed in this study were derived from CHARLS, jointly conducted by the Peking University National School of Development’s Center for Healthy Aging and Development and the Chinese Center for Disease Control and Prevention. The CHARLS dataset is widely cited in the academic community, especially in the areas of aging, health, and labor. It is characterized by its large sample size, longitudinal research design, and comprehensive coverage. The dataset meticulously records information across multiple dimensions, including household composition, employment and retirement status, and health conditions. It includes numerous indicators of labor market participation and a wealth of health status indicators. The CHARLS sample is nationally representative, particularly reflecting the health and labor participation of older adults in China.

In the data preprocessing phase, this study selected the CHARLS dataset organized by Peking University, focusing on the four waves of panel data from 2011, 2013, 2015, and 2018. Considering the research objectives, the study subjects were limited to people aged between 60 and 80 years.

**Figure 1:**
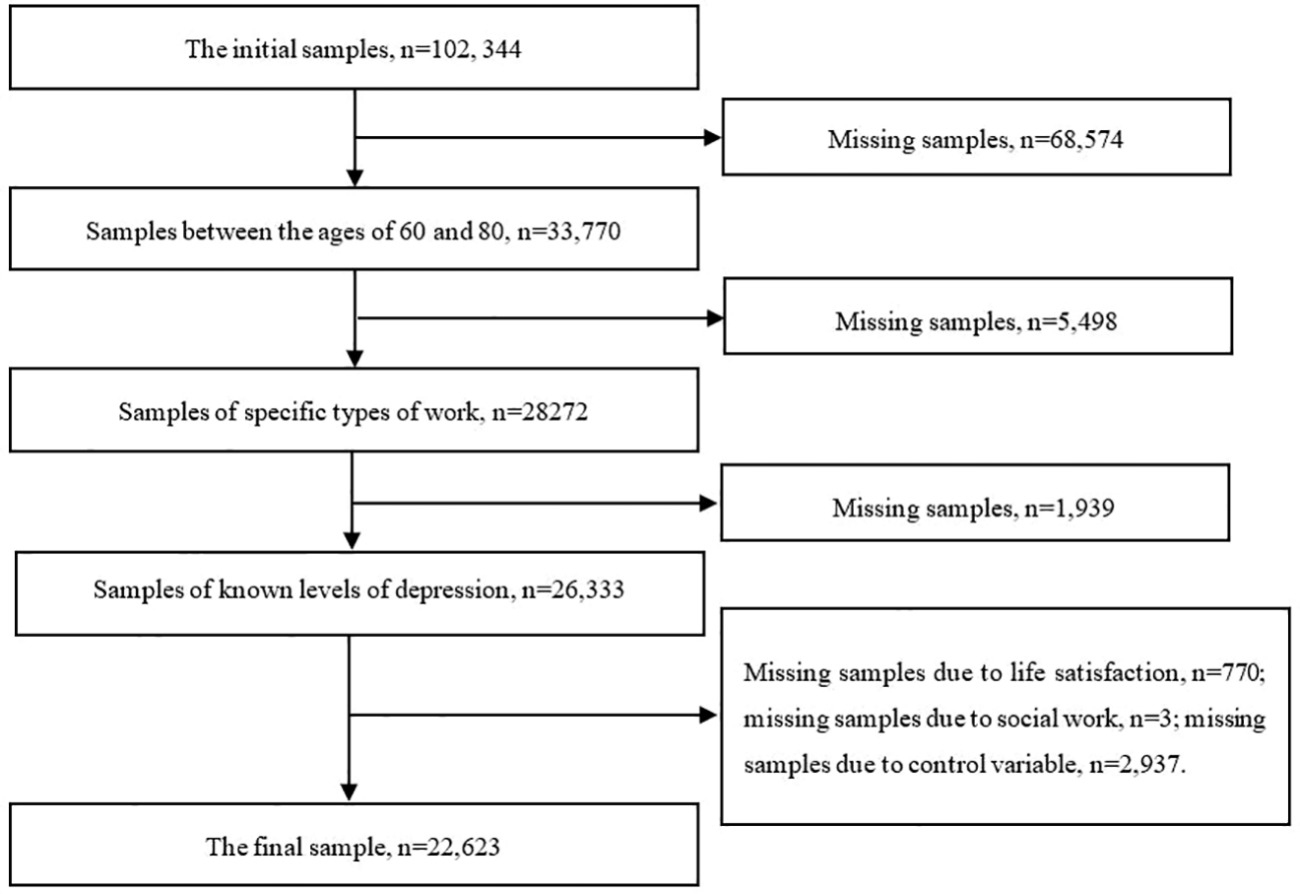
Flow chart for sample selection.

In this study, we employed the method of deleting missing values to handle the data, excluding samples with missing or unanswered key variables. After data cleaning, we obtained 22,623 valid samples. For details on the data cleaning process, please refer to **Figure 1**. All empirical analyses were conducted using Stata 16 software.

### 3.2 Variable measurements

#### 3.2.1 Dependent variable

The dependent variable in this study was depression level, measured using the shortened version of the Center for Epidemiologic Studies Depression Scale (CES-D). The CES-D scale is designed to assess the frequency of current depressive symptoms, focusing on measuring an individual’s depressive mood, and has been shown to have excellent reliability and validity. CES-D10, as a shortened version of CES-D, requires the participants to reflect on the past week and report the frequency of occurrence of 10 depression-related symptoms. The response options include four frequency levels: “Rarely or none (less than 1 day),” “Sometimes (1–2 days),” “Often or about half the time (3–4 days),” and “Most or all of the time (5–7 days).” Each level is assigned a score range of 0–3, resulting in a total depression score range of 0–30, where higher scores indicate a more severe level of depression, suggesting poorer mental health status.

#### 3.2.2 Independent variable

The independent variable in this study was the employment status of older adults. Taking into account China’s situation and to maintain consistency with existing statistical data and research, this study defined people aged 60 or above who were self-employed or retired as study subjects.^6^ Additionally, considering that the physical and psychological capabilities of people aged 80 or above may have significantly declined, resulting in lower labor participation rates, this study opted to exclude this group.

Regarding the definition of employment status, various standards exist in the literature. This study, drawing upon the research by Von Bonsdorff (45) and based on the survey question “What was your work status over the past year?” defined the respondents who answered “Agri self-employed” or “Non-agri self-employed” and were aged 60 years or above as “self-employed older adults” and assigned them a value of 1. Those who answered “retired” and were aged 60 years or above were defined as “retirees” and assigned a value of 0.

#### 3.2.3 Control variables

Drawing on existing research (46), this study selected control variables at both the individual and family levels. For marital status, having a spouse was assigned a value of 1, while not having a spouse was assigned a value of 0. For regular exercise, having a habit of exercising regularly in the past year was valued at 1, and not having such a habit was valued at 0. Drinking frequency was categorized into levels 1–9 based on the frequency of drinking alcohol in the past year, where not drinking alcohol was assigned a value of 1, and drinking alcohol every day was assigned a value of 9. Smoking status was determined by whether one had smoked in the past year, with smoking assigned a value of 1 and not smoking assigned a value of 0. Health status was coded as 1 for those who had been hospitalized in the past year and 0 for those who had not. Personal income, taking the logarithm of the older adults’ income in the past year. Pensions, those with pensions in the past year are assigned a value of 1, and those without pensions are assigned a value of 0. The family-level control variables included child contact, with contacting children assigned a value of 1 and non-contacting children assigned a value of 0, and proximity of children’s residence, with children living near an older adult assigned a value of 1 and children not living near an older adult assigned a value of 0.

It is noteworthy that in this study, to ensure the effectiveness of the control variables, a variance inflation factor (VIF) model was used to test these variables. As shown in **Table 1**, all the control variables had VIF values lower than 10, which means that we could effectively rule out the problem of multicollinearity among the control variables.

**Table 1:** Descriptive statistics.

Variable	N	Mean	Std.Dev.	Min	Max	VIF
Dependent variable						
Depression	22623	8.752	6.509	0	30	
Independent variable						
Work type	22623	0.475	0.499	0	1	
Control variables						
Marriage	22623	0.815	0.389	0	1	1.03
Exercise	22623	0.547	0.498	0	1	1.07
Drinkfre	22623	1.401	2.676	0	9	1.13
Smoke	22623	0.248	0.432	0	1	1.11
Hospital	22623	0.178	0.383	0	1	1.02
Lnincome	22623	0.391	1.79	0	11.813	1.01
Pension	22623	0.935	0.246	0	1	1.01
Childnear	22623	0.79	0.407	0	1	1.40
Childconnect	22623	0.765	0.424	0	1	1.35
Moderating variables						
Social interaction	22623	0.461	0.499	0	1	
Life satisfaction	22623	3.251	0.777	1	5	

#### 3.2.4 Mediating variables

Building on the discussion above, this study considered social interaction and life satisfaction potential mediating mechanisms in the relationship between the employment statuses and depression levels of older adults. Regarding social interaction, this study classified it based on whether the respondents participated in social exchange activities in the past year, with participation in social activities assigned a value of 1 and non-participation assigned a value of 0. Life satisfaction was determined based on the respondents’ answers to the question “Overall, are you satisfied with your life?” Life satisfaction was categorized into five levels, with a value of 1 assigned to those who were not at all satisfied with their lives, and a value of 5 assigned to those who were extremely satisfied with their lives.

### 3.3 Model selection

#### 3.3.1 Fixed effects model

To select the most suitable model, we first conducted a Hausman test to determine the presence of systematic errors, thereby deciding between a fixed-effect model and a random-effects model. The test results showed that Prob > chi2 = 0.0000, with a p-value less than 0.001, which is statistically significant at the 1% significance level. Based on the significant Hausman test results, the fixed-effect model was chosen because it was the more robust estimation method. Furthermore, to fully capture and control for unobservable individual characteristics and time trends, we used a two-way (individual, time) fixed-effect model. This model could help us more accurately estimate the impact of employment status on the depression levels of older adults while controlling for potential biases of unobservable individual and time characteristics. The model was set up as follows:


(1) $$\begin{array}{c} \begin{array}{c} depress_{it}=\alpha_{i}+\alpha_{t}+\beta_{1}worktype_{it}+\gamma X_{it}+\varepsilon_{it} \end{array} \end{array}$$

Where depress_it_ represents the depression level of i-th older adult at t-th time; worktype_it_ indicates the employment status of i-th older adult at t-th time; α_i_ denotes the fixed effect of i-th older adult; α_t_ represents the fixed effect of t-th time; X_it_ stands for other control variables; and X_it_ is the random-error term.

#### 3.3.2 Instrumental variable model

To effectively reduce the bias in regression coefficient estimates caused by omitted variables, this study, while controlling for micro-individual characteristic information in the survey data, further considered the potential bidirectional causality between the employment statuses and depression levels of older adults. To address this potential endogeneity, the study employed the IV model, selecting the number of registered people and the number of students in regular primary schools in various cities for the years 2011, 2013, 2015, and 2018 from the China City Statistical Yearbook as the instrumental variables.

The choice of the aforementioned instrumental variables was significantly rational and effective. First, with the acceleration of urbanization, the increase in the urban registered population generally indicates an economic structural transition, especially the expansion of the service and technology industries. Such changes create new employment opportunities in the labor market, which often require specific skills and pose a potential barrier to many older workers. Therefore, there is a potential link between the increase in the urban registered population and the employment status of older adults, making it suitable as an instrumental variable for analyzing the relationship between older adults’ labor participation and depression levels. Second, the increase in the number of students in urban regular primary schools reflects changes in urban family and demographic structures. An increase in the number of children in a family may lead to higher family living costs, especially in terms of education and childcare. Against the backdrop of increased economic pressure, older adults might participate in the labor force to provide financial support for their families.

Moreover, considering that the two aforementioned instrumental variables are derived from macro-level urban data, they possess higher exogeneity relative to micro-individual characteristics. Overall, these factors support the assumption that the selected instrumental variables are related to endogenous explanatory variables and may be entirely exogenous, thereby providing a solid theoretical basis for this study’s research method.

#### 3.3.3 Moderated mediation model

Drawing from existing research (47) to test potential mediation effects, this study built upon model (1) to construct the following:


(2) $$\begin{array}{c} M_{it}=\delta +\beta_{2}worktype_{it}+\theta_{1}X_{it}+\varepsilon_{it} \end{array}$$

(3) $$\begin{array}{c} depress_{it}=\alpha +\beta_{3}worktype_{it}+\gamma M_{it}+\theta_{2}X_{it}+\mu_{it} \end{array}$$

Where M_it_ represents the mediation variable for the i-th older adult at i-th time; X_it_ stands for other control variables; ε_it_ and μ_it_ denote random error terms; δ, β_3_, and θ_1_ are parameters for the mediation variable equation; and α, γ, β_4,_ and θ_2_ are parameters for the dependent variable equation.

## 4 Results

### 4.1 Descriptive statistics

**Table 1** reports the descriptive statistical results. It can be observed from this table that the sample size is 22,671, with 10,773 self-employed older adults (47.52%) and 11,898 retirees (52.48%). This suggests that self-employment among older adults is not only quite common but may also be related to China’s economic structure and labor market characteristics. Overall, the average depression score for older adults was 8.749, which was close to the threshold of 10, implying that depression among older adults may be quite prevalent. This further highlights the necessity of paying more attention to this issue. Regarding marital status, 81.45% of the older adults in the sample were married or living with a partner. In terms of health habits, about 54.64% regularly engaged in physical exercise, suggesting that most valued their physical health. Additionally, 24.82% of the older adults were smokers, and 17.85% had been hospitalized in the past year. In addition, the income level of the older adults is relatively modest, and the vast majority have pensions. From a family perspective, family relationships are an important part of older adults’ lives. The data show that 76.48% of the older adults in this study had had contact with their children in the past year, and 79% lived close to their children.

### 4.2 Benchmark regression

**Table 2** displays the results of the baseline regression analysis conducted using a two-way fixed-effect model. In Model 1, we directly examined the impact of employment status on the depression levels of older adults. This model indicates that the depression level of self-employed older adults was 0.401 points lower than that of retirees, and this conclusion is statistically significant at the 1% level. Model 2 goes further than Model 1 by incorporating a series of individual-level control variables. After adjusting for these variables, the depression score for self-employed older adults remained 0.322 points lower than that for retirees, with this result being supported at the 5% significance level. In Model 3, we added family-level control variables, but this had a minimal impact on the overall estimation results. This finding strongly suggests that among China’s older population, the depression status of self-employed older adults is significantly better than that of retirees, supporting Hypothesis 1.

**Table 2:** Benchmark regression and robustness test results.

	Model 1	Model 2	Model 3	Model 4	Model 5
	Depression	Depression	Depression	Depression	Depression
Work type	-0.401***	-0.322**	-0.322**	-0.167*	-0.375**
	(0.119)	(0.119)	(0.119)	(0.073)	(0.118)
Marriage		-1.075***	-1.083***	-0.238	-0.938***
		(0.241)	(0.241)	(0.136)	(0.239)
Exercise		-0.225*	-0.227*	0.0189	-0.200*
		(0.100)	(0.100)	(0.063)	(0.099)
Drinkfre		-0.0506	-0.0508	-0.0320	-0.0552
		(0.029)	(0.029)	(0.019)	(0.029)
Smoke		0.0677	0.0668	0.0519	0.0772
		(0.224)	(0.224)	(0.150)	(0.222)
Hospital		0.637***	0.636***	0.191**	0.592***
		(0.120)	(0.120)	(0.073)	(0.119)
Lnincome		-0.0434	-0.0432	-0.00931	-0.0294
		(0.026)	(0.026)	(0.017)	(0.030)
Pension		0.00773	0.00720	0.0426	0.0143
		(0.179)	(0.179)	(0.108)	(0.178)
Childnear			-0.107	-0.0436	-0.127
			(0.142)	(0.090)	(0.141)
Childconnect			-0.0161	0.0460	0.00751
			(0.135)	(0.084)	(0.134)
_cons	9.129***	10.03***	10.14***		10.11***
	(0.102)	(0.415)	(0.439)		(0.452)
N	22623	22623	22623	6771	22167
r2	0.00772	0.0128	0.0129		0.0124

Robust standard errors are in parentheses; *, **, and *** indicate significance at the 10%, 5%, and 1% levels, respectively. Depress, depression.

### 4.3 Robustness tests

The fixed effect model utilized in this study not only controlled for each sample’s intrinsic characteristics that do not change over time, thereby eliminating potential unobserved heterogeneity, but also removed time-specific external shocks experienced by all samples, such as policy changes. However, fixed effect model cannot solve all endogeneity problems, especially reverse causation and so on. Therefore, this study adopted several strategies to improve the robustness of the findings and verify the continuing validity of the main conclusions.

#### 4.3.1 Replace the main effect model

We binarized the dependent variable and attempted to replace the fixed-effect model with a logistic regression model. In Model 4 of **Table 2**, we observed that the average depression symptom score for self-employed older adults was 0.167 points lower than that for retirees, and this result was statistically supported at the 10% significance level.

#### 4.3.2 Elimination of extremes

Considering the potential impact of extreme values or outlier observations on the estimation results, we applied 1% trimming to the data. The estimation results after this processing are noteworthy, as shown in Model 5 of **Table 2**. The processed results show that the average depression score of self-employed older adults was 0.375 points lower than that of retirees, a difference that was verified at the 5% significance level.

#### 4.3.3 Instrumental variable results

**Table 3** shows the two-stage least squares estimation results using the urban registered population and the number of students in urban primary schools as instrumental variables. Specifically, column 1 of the table provides the results of the baseline regression, while columns 2 and 3 display the first- and second-stage regression results of applying the IV model, respectively.

**Table 3:** Instrumental variable results.

	(1)	(2)	(3)
	Fixed effect model	First	Second
Work type	-0.322**		-3.326**
	(0.128)		(1.618)
Iv1		-0.0000527***	
		(0.0000158)	
Iv2		1.16e-7***	
		(1.51e-08)	
Control variables	Yes	Yes	Yes
The F-value of the first-stage		35.15	

Robust standard errors are in parentheses; **, *** indicate significant at the 5%, 1% levels, respectively. Iv1 and Iv2 represent two instrumental variables.

In the first-stage regression, the results indicated a significant correlation between the urban registered population and the number of students in urban primary schools and the employment status of older adults. Furthermore, through a weak instrumental variable test, this study found that the F-statistic value of the first-stage regression was 35.15, significantly higher than the generally accepted threshold of 10. This result strongly indicates that the chosen instrumental variables were not “weak instruments,” meaning they possessed sufficient explanatory power. Additionally, through the Durbin–Wu–Hausman test, with a p-value of 0.0368 (P <0.1), the rationality of the chosen instrumental variables was effectively confirmed, thereby enhancing the credibility of the model’s estimation results.

In the second-stage regression, the estimation results obtained using the IV method showed a significant negative correlation between the employment status and depression levels of older adults. Compared to the baseline regression, the estimated coefficient of older adults’ depression levels increased in absolute value. This finding suggests that the capability of older adults’ employment statuses to lower their depression levels might have been underestimated when endogeneity issues were not considered. Therefore, this study provided a more accurate assessment, showing that the participation of older adults in work activities has a significant positive impact on their mental health status.

### 4.4 Moderated mediation effect

The results indicate that self-employed older adults have a lower likelihood of suffering from depression than retirees. What mechanism accounts for this effect? Based on the aforementioned analysis results, we believe that it is necessary to exclude the impact of pensions on experimental outcomes. Thus, after eliminating the samples without pensions, we further analyzed the potential mediating mechanisms of life satisfaction and social interaction.

Models 1 and 2 in **Table 4** present the regression results, with social interaction as the mediating variable. The results show that when considering the mediating effect of life satisfaction, the regression coefficient of older adults’ depression levels on work status slightly decreases from -0.322 to -0.319. This slight change also suggests that social interaction is a partial mediator. Thus, Hypothesis 2a was confirmed. This may imply that while there are benefits of social interaction on mental health among self-employed older adults, its impact may be limited or interfered with by other more significant factors.

**Table 4:** Mechanism analysis results.

	Model 1	Model 2	Model 3	Model 4
Work type	Social interaction	Depression	Life satisfaction	Depression
	0.0117	-0.319**	0.0260	-0.287*
	(0.011)	(0.119)	(0.017)	(0.117)
Social interaction		-0.231*		
		(0.099)		
Life satisfaction				-1.339***
				(0.063)
_cons	0.433***	10.24***	3.043***	14.22***
	(0.040)	(0.441)	(0.062)	(0.472)
Control variables	Yes	Yes	Yes	Yes
Fixed effect model	Yes	Yes	Yes	Yes
N	22392	22392	22392	22392
r2	0.0126	0.0133	0.0408	0.0485

Robust standard errors are in parentheses; *, **, *** indicate significant at the 10%, 5%, and 1% levels, respectively.

Models 3 and 4 in **Table 4** report the regression results, with life satisfaction as the mediating variable. The results are significant; when considering the mediating effect of life satisfaction, the regression coefficient of older adults’ depression levels on work status adjusts from -0.322 to -0.287. This indicates that life satisfaction indeed plays a mediating role between self-employed older adults and depression. Because the impact of work status on depression level remains significant, this suggests that life satisfaction serves as a partial mediator, confirming Hypothesis 2b. This may be because self-employed people achieve greater work autonomy and better work–life balance, thereby enhancing their life satisfaction. This perspective has also been supported by past research (31).

Considering the potential limitations of the traditional stepwise method in testing mediating effects, to obtain more robust results, we chose to use the bootstrap method to test mediating effects. This method has been regarded as one of the more reliable testing methods in recent years (48). As shown in the test results in **Table 5**, the upper and lower bounds of BootCI did not include 0, and p < 0.01. This indicates that both life satisfaction and social interaction have certain mediating effects between the employment statuses and depression levels of older adults.

**Table 5:** Mediation effect test.

Mediation variable	Effect	Boot standard error	LLCI	ULCI	P-value
Life satisfaction	-0.250	0.091	-0.4302	-0.0701	0.006
Social interaction	-0.340	0.102	-0.540	-0.139	0.001

The upper and lower limits of BootCI were calculated at the significance level of p < 0.01.

### 4.5 Heterogeneity analysis

The relationship between employment status and depression levels may be influenced by various factors. Given the socio-economic and cultural differences between urban and rural areas in China, this study conducted a detailed heterogeneity analysis of the older adults based on their place of residence and *hukou* type. Additionally, considering that different types of self-employment may have varying effects on depression levels among the older adults, this study also distinguishes between the psychological impacts of agricultural and non-agricultural self-employment on older adults.

Models 1 and 2 in **Table 6** reveal the potential impact of the living areas on the depression levels of older adults. Model 1 indicates that, in rural environments, self-employed older adults exhibit lower levels of depression compared to retirees (β_1_= -0.438, p<0.05), possibly due to more frequent social interactions and a stronger sense of social identity brought about by employment in rural areas, thereby helping to alleviate depressive symptoms. Conversely, in urban environments, there is no significant difference in depression levels between self-employed older adults and retirees, possibly reflecting the potential positive effects of diverse social activities and lifestyles on the mental health of older adults in urban areas. Hypothesis 3a was supported.

**Table 6:** Heterogeneity analysis result.

	Model 1	Model 2	Model 3	Model 4	Model 5	Model 6
	Living in rural areas	Living in urban areas	Agricultural *hukou*	Non-agricultural *hukou*	Agricultural self-employment	Non-agricultural self-employment
Work type	-0.438**	-0.0327	-0.360**	-0.236	-0.345**	-0.091
	(0.147)f	(0.208)	(0.134)	(0.320)	(0.125)	(0.374)
_cons	11.44***	8.160***	10.90***	8.356***	10.252***	8.859***
	(0.583)	(0.670)	(0.400)	(0.764)	(0.452)	(0.641)
Control variables	Yes	Yes	Yes	Yes	yes	yes
Fixed effect model	Yes	Yes	Yes	Yes	yes	yes
N	13769	8854	16928	5269	21718	12775
r2	0.0135	0.0143	0.0123	0.0214	0.0133	0.0113

Robust standard errors are in parentheses; **, *** indicate significant at the 5%, 1% levels, respectively.

Meanwhile, Models 3 and 4 in **Table 6** reveal the potential impact of *hukou* type on the depression levels of older adults. Model 3 shows that self-employed older adults with agricultural *hukou* have significantly lower depression levels compared to similar retirees (β_1_= -0.360, p<0.05). This further emphasizes that in rural environments, being self-employed can help reduce depression levels among older adults. However, there is no significant difference in depression levels between self-employed older adults and retirees with urban *hukou*. Hypothesis 3b was supported.

**Table 6**, specifically Models 5 and 6, reveals the potential effects of different types of self-employment on depression levels among older adults. Model 5 indicates that older adults engaged in agricultural self-employment show significantly lower levels of depression compared to retirees (coefficient = -0.345, p<0.05). This suggests that agricultural self-employment, as a meaningful social activity and a source of social support, can significantly improve the mental health of older adults. However, Model 6 shows that non-agricultural self-employment does not have a significant impact on reducing depression levels among older adults (coefficient = -0.091). This may be related to the nature of non-agricultural self-employment, which often involves more isolated work, less social interaction, and greater stress, all of which may negatively affect the mental health of older adults. Therefore, Hypothesis 4 is supported.

## 5 Discussion

This study identified a difference in depression levels between self-employed older adults and retirees, focusing on how social interaction and life satisfaction explain this difference. The findings suggest that self-employed older adults have lower depression levels than retirees. Moreover, in rural areas, self-employed older adults still have lower depression levels compared to retirees. Social interaction and life satisfaction are likely significant factors contributing to notably lower depression levels among self-employed older adults compared to retirees.

### 5.1 The mediating effect of social interaction

Activity theory posits that social participation is crucial for the mental health of older adults (33), with older adults who engage in more social activities likely exhibiting better mental health and lower symptoms of depression (49). Furthermore, research has researches have confirmed that symptoms of depression are associated with less social interaction (50). People with higher levels of depression may have fewer social interactions, and a lack of social interaction and relationships (social isolation) can negatively impact an individual’s physical and mental health, and can even become a significant factor in developing depression (51). Conversely, more frequent and higher-quality interactions can prevent everyday feelings of depression and loneliness, thereby reducing the risk of depression in older adults (52).

Work can increase opportunities for interpersonal interaction and help maintain a positive attitude toward life (53). Upon retirement, older adults may lose work-related goals and roles in social contacts (54), thus losing their social networks among their colleagues (55). However, self-employed older adults, by continuing to work, often have a greater need and more opportunities for social interaction, maintaining a positive spirit and an active social life (31). These factors increase the likelihood of self-employed older adults engaging in more social activities. This finding not only validates activity theory but also explores how work as a form of social participation affects the mental health of older adults.

### 5.2 The mediating effect of life satisfaction

According to activity and role theories, maintaining social roles helps to maintain identity and self-worth among older adults (33). Retirement can be considered a loss of occupational roles for older adults, which may impair their sense of well-being (56). Generally, life satisfaction is an important indicator of subjective well-being (57), and older adults whose sense of happiness is impaired may have lower life satisfaction. Older adults with higher levels of happiness may have a more positive attitude toward life and higher life satisfaction. Additionally, self-employed older adults who continue to work after retirement may have a greater chance of gaining a sense of self-worth, while unemployment or time spent outside the workforce can harm an individual’s sense of self-worth (58). Some studies have also confirmed that having roles and a sense of self-worth may contribute to higher life satisfaction among self-employed older adults (59). Retirees may experience a decrease in subjective well-being and self-worth due to role loss and a lack of work, thereby affecting their life satisfaction.

Lower life satisfaction increases the risk of mental health disorders among older adults (60), including depression (61). Numerous studies have shown a negative correlation between life satisfaction and depression (62). Self-employed older adults with higher life satisfaction are generally more satisfied with their lives and, therefore, may exhibit lower levels of depression compared to retirees.

### 5.3 The urban-rural divide in depression levels among older self-employed individuals

The differences in socioeconomic conditions, infrastructure, public services, resource access, cultural background, and social support systems between urban and rural areas may have varying impacts on the depression levels of self-employed older adults.

Firstly, socioeconomic conditions, along with infrastructure, public services, and resource access, play a crucial role in the differences in depression levels among older self-employed individuals. The underdeveloped socioeconomic conditions, infrastructure, and public services in rural areas not only limit the opportunities for older adults to access resources that alleviate depression (63), but also foster feelings of being forgotten by society or experiencing relative deprivation (38). In this context, self-employment serves as a form of social participation, providing rural older adults with a means to achieve social value and self-identity (64), which helps mitigate their depressive symptoms. In contrast, urban older adults, with better socioeconomic conditions, infrastructure, public services, and access to resources, generally enjoy higher levels of mental health. Therefore, the positive impact of self-employment on urban older adults may not be as pronounced.

Secondly, the cultural differences between urban and rural areas and the corresponding social support systems also play a significant role in the urban-rural disparity in depression levels among older self-employed individuals. The closed and traditional nature of rural culture may limit older adult’s access to sufficient social support and opportunities for social engagement, often leading to feelings of isolation or exclusion (65). Moreover, rural areas lack adequate service infrastructure, which is crucial for promoting older adults participation in social activities (66). Self-employment can increase the social participation of older adults (55), provide a clear sense of purpose in life, and enhance self-worth, all of which contribute to lowering the risk of depression among rural older adults (18). In contrast, urban areas offer not only an open environment with abundant social and recreational activities for older adults (67), but also comprehensive infrastructure and abundant resources that provide full social support and welfare, effectively alleviating symptoms of depression (66). Thus, while self-employment may provide some degree of social interaction and support in the workplace, its effect on reducing depression among older adults in urban areas is less significant.

Additionally, the research results found that the “*hukou*” factor influences the difference in depression levels between self-employed older adults and retirees in rural and urban disparities. In urban areas, and among those with non-agricultural *hukou*, there is no significant difference in depression levels between self-employed older adults and retirees. However, self-employed older adults with agricultural *hukou* exhibit significantly lower depression levels compared to retirees with similar status. This may be due to those with urban *hukou* accessing better medical services and benefits (68) and more pensions (69). Older adults with rural *hukou* face relatively lacking medical services and fewer pensions, which may intensify feelings of deprivation and delay effective treatment for mental health issues. In this context, the self-worth and income provided by self-employment could effectively reduce the risk of depression. Higher levels of medical services and more pension income allow self-employed older adults and retirees with urban *hukou* to maintain a healthier psychological state.

### 5.4 Different types of self-employment in depression levels among older self-employed individuals

Self-employment, as a meaningful social activity, can maintain or even enhance the mental health of older adults. However, the impact of different types of self-employment (agricultural vs. non-agricultural) on depression levels among older adults may vary due to differences in work characteristics and the quality of social support.

In terms of work characteristics, agricultural self-employment involves regular physical activity and contact with nature, and this combination of physical and natural environments provides a way to relieve stress, which positively affects mental health (70). In contrast, non-agricultural self-employment often lacks these elements of physical activity and contact with nature, and the work environment tends to be more isolated and stressful, potentially not offering the same mental health benefits. Regarding the quality of social support, agricultural self-employed individuals are typically embedded within close-knit rural community networks that provide rich emotional and instrumental support, which helps reduce depression levels among older adults (71). On the other hand, non-agricultural self-employed individuals often work and live in urban areas, where they may face higher levels of social isolation and receive less emotional support (72), limiting the opportunity to improve mental health through social interaction. Therefore, compared to non-agricultural self-employment, agricultural self-employment is more effective in alleviating depression among older adults.

## 6 Conclusion

Due to the acceleration of population aging, delaying the legal retirement age has become a global trend. Self-employment is protected and supported by governments worldwide due to its flexible and diverse job characteristics, making it a significant choice for many older adults. This has sparked a high level of concern about the mental health of self-employed older adults and retirees. Is there a difference in depression levels between older adults engaged in self-employment and retirees? Existing research has not provided a clear answer to this question.

This study utilized panel data from CHARLS in 2011, 2013, 2015, and 2018 to examine the impact of self-employment on the depression levels of older adults and to expand and develop activity theory through empirical research. The baseline regression analysis results indicate that the depression level of self-employed older adults is better than that of retirees. Even after controlling for a series of individual and family variables, the results remained similar. The mediation effect test results suggest that lower levels of depression among self-employed older adults may stem from their more frequent social participation and higher life satisfaction. Lastly, the further heterogeneity analysis results indicate that the impact of self-employment on depression levels among older adults exhibits significant differences based on urban-rural distinctions and types of self-employment.

Based on the empirical results presented, a number of policy recommendations are proposed. First, as self-employment has a positive impact on the depression levels of older adults, the government may introduce proactive employment support policies to encourage retired older adults to engage in self-employment. Additionally, it is crucial to improve employment security for older adults, such as enhancing their work injury and medical insurance coverage and providing public employment services to alleviate the burden of self-employment on older adults. Second, the value needs of older adults on a psychological level must be addressed. This can be achieved through community activities and increasing venues for older adults’ activities, thereby expanding their social participation, enhancing their life satisfaction, and achieving the goal of lowering the depression levels of older adults. Furthermore, given the positive role of self-employment in alleviating depression among rural older adults compared to their urban counterparts, more emphasis must be placed on promoting self-employment among the former to foster the balanced development of self-employment between urban and rural areas. Active promotion of the significant contribution of self-employment to society must be pursued to achieve beneficial, healthy aging. Finally, considering the varying effects of different types of self-employment on the depression levels of older adults, it is essential to strengthen support and encouragement for rural older adults to engage in agricultural self-employment, utilizing its unique work characteristics and community support environment to promote mental health. At the same time, urban areas should improve the social support networks and work environments for non-agricultural self-employed individuals, fostering balanced development of self-employment for older adults across both urban and rural settings.

Despite conducting a thorough empirical analysis and discussion, this study has some limitations. First, based on the requirements for data quality and analytical accuracy, we used the deletion of missing values method to exclude samples containing key variables that were missing or refused to be answered. Although this step is crucial for ensuring the reliability of a study, it also introduces specific limitations, such as potential biases in the experimental results. Thus, we plan to adopt more comprehensive data processing methods, such as multiple imputation, in subsequent research to minimize the impact of sample exclusion, thereby enhancing the accuracy of the analysis and the reliability of the results. Second, although we were able to identify the labor statuses of older adults at the time of the survey, due to data limitations, we could not accurately distinguish between older adults who had always been self-employed and those who transitioned from other employment statuses to self-employment. This limitation may have affected our comprehensive understanding of the diversity of self-employment status among older adults. In the future, we will consider using more diverse data sources and adopting more advanced research methods, such as life cycle analysis or dynamic panel models, to track historical changes in the labor statuses of older adults.

## Data Availability

The original contributions presented in the study are included in the article/supplementary material. Further inquiries can be directed to the corresponding author.

## Footnotes

1 Depression. World Health Organization, https://www.who.int/zh/health-topics/depression#tab=tab_1

2 Depression. World Health Organization, https://www.who.int/zh/health-topics/depression#tab=tab_1

3 Organization of Economic Co-operation and Development/European Commission (2021), The Missing Entrepreneurs 2021: Policies for Inclusive Entrepreneurship and Self-Employment, OECD Publishing, Paris, https://doi.org/10.1787/71b7a9bb-en.

4 National Bureau of Economic Research, https://www.nber.org/brd/self-employment-older-ages

5 Hukou refers to China’s residency registration system, which determines access to social benefits and rights based on an individual’s registered place of residence. This unique system plays a significant role in Chinese society and administration

6 National Bureau of Statistics of China, http://www.stats.gov.cn/zs/tjws/tjbz/202301/t20230101_1903949.html.
